# Supplementary material for: The impacts of preoperative frailty on readmission after cardiac implantable electrical device implantation
Source: PLoS One. 2022 Nov 3;17(11):e0277115. doi: 10.1371/journal.pone.0277115 (PMC9632810; doi:10.1371/journal.pone.0277115)
Supplement: Attachment — Submitted filename: Response to Reviews.docx [file pone.0277115.s001.docx]

Response to Reviewers’ Comments

We are grateful for being given the opportunity to resubmit our revised manuscript entitled, “The impacts of preoperative frailty on readmission after cardiac implantable electrical device implantation.”

We have incorporated changes that reflect the detailed suggestions made by the reviewers and believe that the revised manuscript has significantly improved as a result.

We also hope that the responses provided below satisfactorily address all the issues and concerns of the reviewers and the editor, and that the manuscript will now be acceptable for publication in PLOS ONE.

We have provided point-by-point answers below to the questions and comments presented in your letter dated July 15.

ACADEMIC EDITOR comment #0

The manuscript is affected by several shortcomings that do not help to support the conclusions. A paragraph dedicated to limitations of the study should be emphasized. Moreover, the gender impact should be well addressed.

Response for Editor comment #0

We appreciate your concern about the issue, which is completely appropriate and important. Frailty and heart failure in women are related. However, our study population included many male participants who belonged to the heart disease population, and there was no difference of gender between the frailty and non-frailty groups. Thus, the effect of gender is considered minimal.

We have added the following sentences to the “Study limitation section” about the gender impact. We have pointed out other limitations of the study too. Please review the comments section of the reviewer for the same.

“The most noteworthy problem was the differences in patient characteristics between the frailty and non-frailty groups and rates of sex, in addition to the small number of patients and events.” (Page 24–25, lines 329–331)

“Another difference in patient characteristics is sex. Female frailty patients with HF have been shown to be at a higher risk of readmission [30]. The patients in our study were predominantly male. Thus, we cannot rule out that sex may have influenced readmission.” (Page 25, 336–338)

“Patients’ sex has important research implications for frailty. For example, female frailty patients with HF have been shown to be at a higher risk of readmission [30]. However, in this study, the percentage of women was low as the subject of this study was heart disease. In addition, since mFI is an index that does not include sex, we consider sex to have had little effect on this study. The results of our multivariate analysis showed that frailty was significantly associated with readmission within a one-year period.” (Page 20, lines 241–246)

Editor comment #1

1. Please ensure that your manuscript meets PLOS ONE's style requirements, including those for file naming. The PLOS ONE style templates can be found at
   <https://journals.plos.org/plosone/s/file?id=wjVg/PLOSOne_formatting_sample_main_body.pdf> and 
   <https://journals.plos.org/plosone/s/file?id=ba62/PLOSOne_formatting_sample_title_authors_affiliations.pdf>.

Response for editor comment #1

Thank you very much for your suggestions and valuable comments.

Our manuscript was adapted to your journal’s style with great care.

Editor comment #2

2. Please provide additional details regarding participant consent. In the ethics statement in the Methods and online submission information, please ensure that you have specified (1) whether consent was informed and (2) what type you obtained (for instance, written or verbal, and if verbal, how it was documented and witnessed). If your study included minors, state whether you obtained consent from parents or guardians. If the need for consent was waived by the ethics committee, please include this information.

If you are reporting a retrospective study of medical records or archived samples, please ensure that you have discussed whether all data were fully anonymized before you accessed them and/or whether the IRB or ethics committee waived the requirement for informed consent. If patients provided informed written consent to have data from their medical records used in research, please include this information.

Response for editor comment #2

We are grateful for your valuable suggestions and comments.

As this is a retrospective study, the requirement of informed consent from patients was waived and an opt-out method was adopted, which allows patients to express their desire to not participate. It was approved by both ethics committees. We have added the following sentences acknowledging the same in Methods on page 8, lines 139–143)

“The requirement of informed consent from patients was waived, and we adopted an opt-out method, which allows patients to express their desire to not participate. It was approved by both ethics committees. The information regarding the use of medical record data for the study and the opt-out method is presented on our hospital's website.”

Editor comment #3

3. Thank you for stating the following in the Competing Interests section: 
Dr. Kato and Dr. Ikeda received grant support from Boston Scientific, Abbot, paid to his institute.
Dr. Makita received payment from Daiichi Sankyo Company, Otsuka pharmaceutical, and Fukuda denshi for lectures, presentations, speakers bureaus, manuscript writing, or educational events for his institute.

Please confirm that this does not alter your adherence to all PLOS ONE policies on sharing data and materials, by including the following statement: "This does not alter our adherence to  PLOS ONE policies on sharing data and materials.” (as detailed online in our guide for authors <http://journals.plos.org/plosone/s/competing-interests>).  If there are restrictions on sharing of data and/or materials, please state these. Please note that we cannot proceed with consideration of your article until this information has been declared.

Please include your updated Competing Interests statement in your cover letter; we will change the online submission form on your behalf.

Response for editor comment #3

Thank you very much for making this important suggestion.

We have confirmed our adherence to the policy of PLOS ONE and the COI statement has been modified accordingly. The statements regarding COI have been altered in the cover letter too.

Editor comment ＃4

4. In your Data Availability statement, you have not specified where the minimal data set underlying the results described in your manuscript can be found. PLOS defines a study's minimal data set as the underlying data used to reach the conclusions drawn in the manuscript and any additional data required to replicate the reported study findings in their entirety. All PLOS journals require that the minimal data set be made fully available. For more information about our data policy, please see <http://journals.plos.org/plosone/s/data-availability>.

"Upon re-submitting your revised manuscript, please upload your study’s minimal underlying data set as either Supporting Information files or to a stable, public repository and include the relevant URLs, DOIs, or accession numbers within your revised cover letter. For a list of acceptable repositories, please see <http://journals.plos.org/plosone/s/data-availability#loc-recommended-repositories>. Any potentially identifying patient information must be fully anonymized.

Important: If there are ethical or legal restrictions to sharing your data publicly, please explain these restrictions in detail. Please see our guidelines for more information on what we consider unacceptable restrictions to publicly sharing data: <http://journals.plos.org/plosone/s/data-availability#loc-unacceptable-data-access-restrictions>. Note that it is not acceptable for the authors to be the sole named individuals responsible for ensuring data access.

We will update your Data Availability statement to reflect the information you provide in your cover letter.

Response for editor comment #4

Thank you very much for your important comments.

We have reviewed the Data Availability statement and added it accordingly. We have also added the Data Availability statement in the cover letter.

5. Review Comments to the Author

Please use the space provided to explain your answers to the questions above. You may also include additional comments for the author, including concerns about dual publication, research ethics, or publication ethics. (Please upload your review as an attachment if it exceeds 20,000 characters)

Response to the Reviewers’ Comments

Reviewer #1:

Comment 1 of Reviewer#1

 I would congratulate to the patients for this good study, investigating whether preoperative frailty could influence readmission rates among patients with CIED over a one-year period following implantation. Results are extremely interesting, here you find suggestions in order to improve the manuscript:

Introduction: authors state that "Currently, three highly therapeutic cardiac implantable electrical devices (CIED) are in common usage, including pacemakers (PM) for patients with bradycardia, implantable cardioverter defibrillators (ICD) for the prevention of sudden death, and cardiac resynchronization therapy (CRT) for patients with heart failure (HF) and cardiac dyssynchrony" Authors should also discuss alternative techniques for left ventricular pacing in cardiac resynchronization therapy including: surgical access representing the most frequently used as a second choice by either minithoracotomy or especially the video-assisted thoracoscopy (DOI: 10.1111/pace.12320), His pacing (DOI: 10.1111/pace.14336) or Left Bundle Branch Pacing (DOI: 10.3389/fcvm.2021.630399). Please cite this points including 3 fundamental references

Response for Comment 1 of Reviewer#1

Thank you for your insightful comments.

As you pointed out, device treatments for heart failure such as CRT and conduction system pacing have progressed in recent years. In this study, there were no cases in which conduction system pacing was performed; hence, we believe that it had no effect on our results. However, it is important to mention the content of these technologies as they contributed to the development of heart failure therapy.

We have added the following sentences to the Introduction:

“In recent years, a new method of left ventricular lead implantation for CRT using mini-thoracotomy or video-assisted thoracoscopy has been reported. In addition, conduction system pacing (e.g., his bandle pacing and left bandle branch pacing) has been presented as an advanced alternative to CRT; however, these methods do not guarantee perfect effect of heart failure therapy [1–3].” (Page 4, lines 57–61)

“No patients underwent His pacing in this study.” (Page 9, lines 152–153)

Comment 2 of Reviewer#1

Discussion: "In general, patients with PM and ICD have a lower rate of HF readmission and mortality when compared to CRT patients" However, among CRT, authors should also discuss about its potential effects on functional performance and cognition, two determinants of disability, frailty development and survival. Fumagalli et al demostrated that CRT may be associated with higher functional and cognitive profile (DOI: 10.1016/j.ijcard.2016.06.001). These finding could let us hypothesize a powerful effect of treatment to frailty development and may impact the patient population; please discuss in discussion as well as limitation sections

At the same non responders to CRT should be considered. Please discuss and amplify discussion

Response for Comment 2 of Reviewer#1

We are grateful to the Reviewer for this comment that guided us in discussing this important point, which was missing in the previous version of the manuscript.

As you pointed out, this study lacked the perspective of CRT as a device for heart failure therapy. It seems necessary to distinguish CRT from PM as a device for bradycardia and ICD as a device for tachycardia. When we added an investigation of non-responder rate of CRT, we found a tendency for there to be more non-responders in the frail group, although there was no significant difference. Cognitive function was not investigated in detail in this study, but cognitive function may also attenuate the effects of CRT in the frail population.

We have updated the results and discussion sections by adding sentences regarding this idea. Please confirm their validity.

“CRT non-responders (defined as improvement in left ventricular end-systolic volume of under 15% from the baseline using echo cardiography at 6 to 12 months after CRT) tended to be more frequent in the frailty group (non-frailty group vs. frailty group = 33.3% vs. 45.5%, p=1.00).” (Page 11, lines165–168)

“The potential impact of physical and cognitive dysfunction on CRT is higher than those of PM and ICD. Frailty has been indicated as being an effect on readmission in CRT patients [13]. In particular, it has been reported that frailty was more frequent in non-responders to CRT [31]. Furthermore, there is literature suggesting a cognitive impairment in CRT patients [32]. Cognitive dysfunction has been an independent factor that increases readmission in heart failure [33]. In this study, only 16.8% of the current participants had CRT. When we added an investigation of the CRT non-responder rate in frailty and non-frailty group, we found that the rate of non-responders tended to be high in the frailty group, but there was no significant difference. Non-responders of CRT may be a cause of readmission for heart failure. CRT non-responders and frailty are covariates and difficult to separate because of a small number of objects. In addition, cognitive function was not investigated in detail in this study, but it may also attenuate the effects of CRT in the frailty population.” (Page 20–21, lines 256–267)

We have also added the following sentences to the Study limitation section:

“This indicates the need for postoperative evaluations aimed at determining whether preoperative or postoperative frailty and cognitive dysfunction impact readmission.” (Page 25–26, lines 348–350)

We have added Non-responders to the CRT section in Table 1 and an asterisk has also been added to define a Non-responder. (Page 9–10, Table 1)

Comment 3 of reviewer #1
A nice figure showing mechanisms involved in frailty is definitely welcome

Response for Comment 3 of reviewer #1

We thank the reviewers for their constructive suggestion, which helped us to remarkably improve our manuscript.

In our opinion, some CIED patients are prone to frailty. In particular, those with frailty develop a vicious circle owing to the correlation between muscle mass loss, frailty, and heart failure and may suffer heart failure.

We have created a chart that shows the correlation between frailty and heart failure in CIEDs patients. Moreover, we have updated the discussion by adding sentences conveying this point.

“Frailty, muscle, and CHF have a dynamic interrelation (Fig 4). Frailty and muscle are affected by activities of daily life, sensory impairment, cerebrovascular accident, peripheral vascular disease, and TIA. Some CIED patients with a background of cardiac disease are more prone to frailty. Hypertension, diabetes mellitus, myocardial infarction, angina, PCI/cardiac surgery, and chronic obstructive pulmonary disease cause frailty and are also direct causes of heart failure. Moreover, these induce organic disorder, which causes heart failure. Patients with frailty prior to CIED implantation have reduced activity, muscle strength, and power, which could further exacerbate heart failure after implantation. Reduced muscle mass or muscle function decreases muscle pump function and increases the risk of heart failure. Heart failure not only reduces cardiac function, but also contributes to reduced exercise tolerance, making the patient more susceptible to fatigue, which further contributes to heart failure symptoms.” (Page 21–22, lines 270–280)

Kindly also note the figure legend that we have included in the manuscript to follow your valuable suggestion.

“Fig 4. Correlation chart for frailty, heart failure, CIED patients, and other mFI factors.

Frailty and muscle are affected by ADL, sensory impairment, CVD, PVD, and TIA. Frailty and CHF are affected by hypertension, diabetes mellitus, MI, anginal pain, PCI, and cardiac surgery. Muscle and frailty are associated with decreased muscle mass and strength and decreased activity. Muscle and CHF are related to exercise tolerance and muscle pump action. Frailty and CHF are related to cardiac dysfunction and fatigue.

Abbreviations are the same as in Table 1 and Figure 1.” (Page 22, lines 282–289)

Comment of Reviewer #2:

 In this paper Authors describe how frailty influences the outcome after placing a cardiac stimulator.
The interest of the paper and its relevance are jeopardized by the limited numbers of patients and of events.
Otherwise the paper highlight the importance to take in account the frailty in any procedure to avoid futile treatment.
In general the article is well written but of scarse relevance.

Response for comment of Revewer ＃２

We agree with the reviewer.

The small number of cases and few events are grave limitations of our study.

Additionally, since mFI is an index that includes heart diseases such as heart failure, myocardial infarction, and history of coronary intervention, it tends to be higher in CIED patients.

Therefore, we must admit that what you mentioned resonated with what we had originally thought.

The problem has already been mentioned in the Discussion section under the title “Usefulness and bias of mFI” and in the Limitation section.

However, it is worth noting that the high mFI group had a higher hospitalization rate even among the CIED patients.

As you pointed out, it may be difficult to find relevance between frailty and CIED patients.

However, it is unusual for heart failure to be the cause of all the readmissions in CIEDs, and this result could be specific to CIEDs with frailty.

Our study is statistically weak; however, we believe it is worth reporting because this is one of the few studies that focused on CIED patients with frailty.

We have redrafted the Study limitation section to establish a clearer focus:

“The most noteworthy problem was the differences in patient characteristics between the frailty and non-frailty groups, rates of sex, in addition to the small number of patients and events” (Page 24–25, lines 329–331).

We have added the following sentences to the subsection entitled “Usefulness and bias of mFI” in the Discussion section: “Therefore, previous studies may have rather evaluated frailty. Patients with high mFI seemed to be prone to readmission in the hospital with or without a CIED. Future studies should increase the number of patients and consider the differences between patients with and without a CIED.” (Page 23, lines 308–311)

We have also added the following sentences to the Study limitation section.

“Hence, future studies should predict readmission using the mFI among larger samples, controlling for patient characteristics. Furthermore, it may be necessary to develop an advanced index that excludes the association with heart disease.” (Page 25, 338–341)

In addition, we have included sentences to the subsection titled as Clinical implications. “This study produced three important results for consideration in the clinical context. First, we found that the mFI could safely be used to predict one-year readmission prior to CIED implantation. Second, we believe that our results could help avoid futile treatment. Third, preoperatively applied mFI can be used to determine the risk of readmission early in the rehabilitation process.” (Page 24, lines 315–319)

Other parts independently modified by the author

- We have renumbered the references to reflect the addition of reference papers. The added references are 1, 2, 3,30, 31, 32, 33 and 34.
- Abbreviations in Tables 1, 2, 3, 4, 5 and Fig 2 have been rewritten to ensure ease of reading.
- We have added one word for readability in the Methods section. ” Based on previous studies, those with two or fewer mFI items were placed into the non-frailty group, while those with three or more items were placed into the frailty group. The primary outcome was defined as follows: readmission for any cause deemed to require inpatient care within one year of treatment; the maximum follow-up period was regulated per the year.” (Page 6, 109–113)
- We have made an error in the use of the word and are correcting it in the Statistical analysis section. “Based on the outcomes of previous studies, we selected the following dependent variables: sex [19, 20]” (Page7, lines119–120)
- We have appended words for readability.” More precisely, it is a modified version of the R commander which is designed to add statistical functions that are frequently used in biostatistics [29].”(Page7–8, lines132–134)
- We were missing a word, so we added it in the Results section. ” As shown, the mean age was 75.8 ± 6.2 years, with 38 (37.4%) female participants. Of all participants, 64 (63.4%) had PM, 20 (19.8%) had ICD, and 17 (16.8%) had CRT.” (Page8–9, lines150–152)
- There was a typographical error in Fig 1, which we have corrected. ” 121 patients with newly implanted CIEDs (PM, ICD, or CRT) or who underwent battery replacements at our hospital between May 2016 and January 2020”
- A typographical error was made in the legends section of Figure 3, which has been corrected. “Fig 3. Kaplan-Meier curve comparing time to development for uncompensated heart failure in patients with cardiac implantable electrical devices (CIED), as classified according to frailty condition, adjusted HFrEF, and CIED type. (Page 14, 204–206)
- We have added to the subtitle. “Relationship between frailty and readmission in CIED patients” (Page 19, lines 231)
- We have changed the wording for readability in Discussion section. “While this study used the mFI to determine frailty, previous studies have used the Fried method [34]. (Page22, lines 293–294)
